# Supra-skirtal leakage following subacute valve migration after TAVI

**DOI:** 10.1007/s12928-026-01281-3

**Published:** 2026-05-12

**Authors:** Yusuke Morita, Akihiro Endo, Shuntaro Tanaka, Seita Yamasaki, Hirotomo Sato, Kazuaki Tanabe

**Affiliations:** 1 Division of Cardiology, Shimane University Faculty of Medicine, 89-1 Enya- cho, Izumo, 693-8501 Japan; 2 Division of Cardiology, Masuda Red Cross Hospital, i 103-1 Otoyoshi- cho, Masuda, 698-0003 Japan

An 89-year-old man with symptomatic severe aortic stenosis underwent transcatheter aortic valve implantation (TAVI). Preprocedural computed tomography (CT) demonstrated an annular area of 410 mm². Moderate calcification was observed in the non-coronary cusp, while the right-coronary cusp had mild calcification, and the left-coronary cusp had almost no calcification (Fig. 1A-B). A 23-mm SAPIEN 3 Ultra RESILIA valve (Edwards Lifesciences, USA) was implanted via a transfemoral approach at nominal volume (Fig. 1C). Mild paravalvular leakage (PVL) persisted after deployment, and single post-dilatation reduced PVL to trivial. No migration was observed on transthoracic echocardiography (TTE) immediately after TAVI (Fig. 1D).

Several hours later, TTE revealed unexpected migration toward the left ventricle with moderate PVL progression. Ten days later, TTE and CT demonstrated further migration and severe aortic regurgitation caused by supra-skirtal leakage (SSL) (Fig. 1E-G and Video S1). As a consequence of persistent SSL, hemoglobin decreased from 13.5 g/dL to 7.6 g/dL, lactate dehydrogenase increased to 2272 U/L, and haptoglobin levels decreased, findings consistent with hemolytic anemia.

Given progressive migration and hemolytic anemia, TAV-in-TAV was performed on day 18. Despite an area of 352 mm² of the initial valve, a 26-mm valve with − 2 mL underfilling was selected based on the native annular area and migration (Fig. 1H). A 26-mm SAPIEN 3 Ultra RESILIA valve was implanted at the appropriate annular level, intentionally positioning the new prosthesis above the displaced initial valve (Fig. 1I-L). Aortic regurgitation improved to trivial, and hemolytic markers normalized (Video S2). The patient was discharged 1 week later, and no recurrence of hemolysis during the 6-month follow-up.

This case illustrates that subacute valve migration can occur after an optimal acute result, particularly in anatomies with asymmetric and limited annular calcification, where relatively mild calcification in the right- and left-coronary cusps contrasts with more pronounced deposits in the non-coronary cusp. In such settings, undersizing or insufficient expansion may reduce anchoring forces, leading to prosthesis migration from the annular plane.

**Fig. 1:**
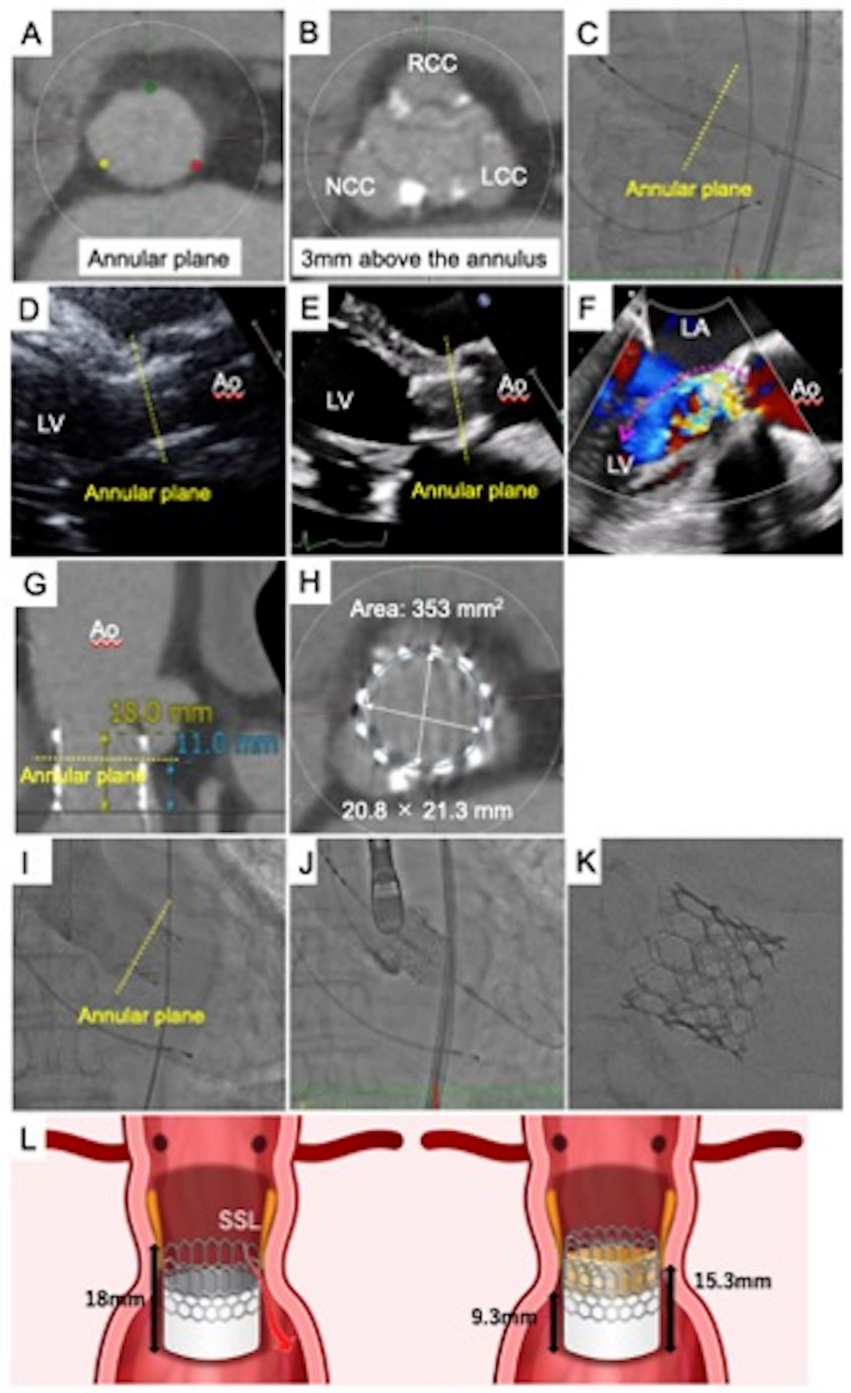
(**A**,** B**) Preoperative CT. (**C**) First-valve implantation. (**D**) TTE showing no migration immediately after TAVI. (**E-G**) TTE (**E**) and CT (**G**) demonstrate valve migration, while transesophageal echocardiography. (**F**) reveals an SSL from the left-coronary cusp (arrow). (**H**) CT measurements after first-valve implantation. (**I**) Aortic angiography before second-valve implantation. (**J**) Second-valve implantation. (**K**) Magnified view following second-valve implantation. (**L**) Schematic of SSL mechanism (left) and sealing by TAV-in-TAV (right). The 23-mm SAPIEN 3 frame, skirt, and risk-plane heights are 18, 9.3, and 15.3 mm, respectively.

Once migration toward the left ventricle occurs, exposure of the stent frame above the sealing skirt creates a high-velocity regurgitant pathway, resulting in SSL and subsequent hemolysis. Thus, heterogeneous cusp calcification, particularly a minimally calcified cusp, may predispose to migration. TAV-in-TAV is an effective therapeutic strategy [1].

## Supplementary Information

Below is the link to the electronic supplementary material.

Supplementary Material 1

Supplementary Material 2
